# Preventing pressure injury in open‐heart surgical patients: A systematic review

**DOI:** 10.1002/hsr2.1148

**Published:** 2023-03-17

**Authors:** Hamed Taghiloo, Abbas Ebadi, Yaser Saeid, Alireza Jalali Farahni, Atefeh Davoudian

**Affiliations:** ^1^ MSc in Operating Room, School of Nursing Baqiyatallah University of Medical Sciences Tehran Iran; ^2^ Behavioral Sciences Research Centre, Life Style Institute, School of Nursing Baqiyatallah University of Medical Sciences Tehran Iran; ^3^ Trauma Research Center and Faculty of Nursing Baqiyatallah University of Medical Sciences Tehran Iran; ^4^ School of Medicine, Atherosclerosis Research Center Baqiyatallah University of Medical Sciences Tehran Iran; ^5^ Deputy of Research and Technology Zanjan University of Medical sciences Zanjan Iran

**Keywords:** cardiac surgical procedures, pressure ulcers, prevention and control, systematic reviews, thoracic surgery

## Abstract

**Background:**

Cardiac surgical patients are highly prone to developing surgery‐related Pressure injuries (PIs). Prevention of PIs is an important patient safety priority in healthcare settings and patients care. So the aim of this study is to detect the effectiveness of prevention strategies to decrease PIs prevalence and incidence in patients undergoing open heart surgery.

**Method:**

We identified studies through Web of Science, Scopus, PubMed, Cochrane, and ProQuest databases from inception through September 2022 with restrictions on the English language. Cochrane RoB 2, JBI, and NIH checklist were carried out as critical appraisal Tools to investigate the studies risk of bias. Finally, 10 studies with a total sample No. 1348, which fulfilled eligibility criteria were included in final systematic review.

**Result:**

Most common interventions investigated in included studies were addressing impairments skin care which included the use of multilayer silicone foam, Care bundle and multiple intervention programs, alternative head inflatable pads, pressure‐reducing foam mattresses, and electric bed frames as the effective PIs Prevention (PIP) strategies. While repositioning is one of the important causes mentioned in most PIP protocols, there was not adequate evidence to recommend any special turning regimens for PIP.

**Conclusion:**

Given current evidence, multilayer silicone foam, Care bundle and multiple intervention programs, alternative inflatable head pads, pressure‐reducing foam mattresses, and electric bed frames are effective strategies to prevent pressure ulcers. Further investigations are needed to specify the cost‐effectiveness of mentioned strategies and RCTs to determine other PIP strategies such as repositioning and mobilization, nutritional supplementation, creams, and co‐interventions effects.

## 1 INTRODUCTION

Pressure injuries (PIs) known as pressure ulcers, are one of the costliest medical problems and physically debilitating for patients. PIs are a common side effect of many high‐acuity surgeries, and it has known as a major comorbid event due to cardiac surgery.^1^ cardiac surgery patients are considered one of the most at‐risk patient populations, with incidence rates reported as high as 24.06%.^2^ PIs are potentially preventable but frequently occurring adverse events in hospitalized patients.^3^ Prevention of PIs is an important patient safety priority in healthcare settings because PIs make a significant independent contribution to the excess patients’ length of hospitalization.^4^ In addition, patients must pay a significant additional cost for PIs treatment.^5^ A systematic review found out Female sex, diabetes, advanced age, Duration of surgery and preoperative serum albumin level as the risk factors for developing PI in cardiac surgical patient that it needs to consider for PI prevention before and during the surgery.^2^ A literature review also indicates that there is a variety of clinical studies on the use of dressings in the prevention of PI, such as hydrocolloids, foams, and films during surgery.^6^ Some other studies proved that the incidence of PI can be reduced by using the multi‐layered silicone foam, because of its ability to reduce friction, pressure forces, and transferring shear away from critical spots. It can also reduce the costs.^6^, ^7^, ^8^ The 2019 National Pressure Injury Advisory Panel (NPIAP, old NPUAP) guideline recommends using a pressure redistribution support surface on the operating table, such as a viscoelastic polymer pad, in patients undergoing surgery who are at the risk of developing pressure injuries; meanwhile, a soft silicone multilayered foam dressing is recommended in patients at risk of developing pressure injuries, but evidence for surgical patients is insufficient^9^ and to our knowledge, there has been no systematic review conducted on the effectiveness of multicomponent PIs prevention programs, their components, and the strategies used to implement such programs in cardiac surgical patients. So we conducted a systematic review with the aim of detecting the effectiveness of PIs prevention strategies and interventions in reducing PIs prevalence and incidence in patients undergoing open‐heart surgery.

## 2 METHODS

### 2.1 Search strategy and information sources

We accomplished a systematic review, registered on PROSPERO (ID: CRD42022364468). This systematic review was performed based on the Preferred Reporting Items for Systematic Reviews and Meta‐Analysis (PRISMA) guidelines.^10^ We formulated the research question according to the “PICO” format. Which interventions (single or bundled) are effective in preventing pressure injury in the open‐heart surgical patients? Our review was conducted by reviewing the related literature in Web of Science, Scopus, PubMed, Cochrane Central Register of Controlled Trials (CENTRAL), and ProQuest electronic databases without publication period restriction (up to September 2022) for English language clinical trials. "MeSH and non‐MeSH keywords used to find" related studies were “Thoracic Surgery,” “Coronary Artery Bypass,” Embolectomy, “Heart Surgery,” “Cardiac Surgery,” “Cardiac Surgical Procedure,” “Heart Transplantation,” “Cardiac Valve Annuloplasty,” “Pressure Ulcer,” Bedsore, “Pressure Sore,” “Decubitus Ulcer,” “Pressure injury,” “Prevention” and “Control”). Additional searches were conducted on conferences, registries, Google Scholar, and government websites. The reference lists of studies were also checked.

### 2.2 Study selection process

Two researchers screened the abstracts of all retrieved studies to determine potentially relevant studies for this review. Then, the studies’ full text was independently assessed by two researchers to verify the qualified articles to be included. Any disagreement was referred to third researcher if they haven't been settled by discussion. The inclusion criteria were Randomized Controlled Trials (RCT), quasi‐experimental and nonrandomized trials, pretest and posttest (before and after), and the studies that included the efficacy of preventive strategy on PI incidence in patients undergoing open‐heart surgerie—PI developed during surgery—(≥18 years old).

The exclusion criteria were the reviews, letters, editorials, and retrospective studies, lack of addressing the preventing PI, PI developed after surgery at critical care unit, being a duplicate, low quality, and high risk of bias according to critical appraisal tools.

### 2.3 Quality assessment of the studies included

The bias risk assessment was carried out using Cochrane Risk‐of‐Bias tool for randomized trials (RoB 2),^11^ Joanna Briggs Institute (JBI) Checklist for Quasi Experimental Appraisal tool,^12^ and The National Institutes of Health (NIH) quality assessment tool for before‐after (pre–post) study with no control group.^13^ RoB 2 tool is structured into five domains for individually randomized trials^1^: bias arising from the randomization process^2^; bias due to deviations from intended interventions^3^; bias due to missing outcome data^4^; bias in measurement of the outcome^5^; bias in selection of the reported result. JBI checklist has five questions, which use to determine the quality of Quasi‐experimental studies (nonrandomized) and NIH tool has 12 questions for quality assessment of before–after (pre–post) study without a control group.

### 2.4 Data extraction

Two researchers extracted the data by using a pre‐specified form. The extracted data included the author's name, year of publication, study design, sample size, setting, intervention, result, conclusion, and so forth.

### 2.5 Synthesis method

We summarized the included studies’ characteristics, the components of the PIs prevention strategies, programs and intervention used, and the studies outcomes. Due to variations of strategies and interventions that were implemented, and the difference between outcome definitions and measures, we were unable to perform meta‐analyses.

## 3 RESULTS

### 3.1 Study selection

**Figure 1:**
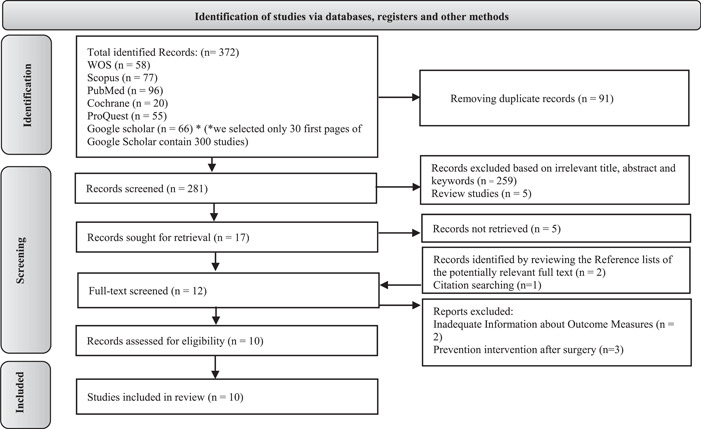
PRISMA flow diagram showing the selection process of the qualified articles.

The PRISMA flow diagram^10^ of the studies’ selection process is shown in Figure 1. Three hundred and seventy‐two studies were retrieved from electronic database searches. The title and abstract of 281 studies were reviewed after removing the duplicates. Two hundred and sixty‐four studies were excluded and 17 articles were left to full‐text review (five full‐text were not retrieved). Three additional studies were found by searching the reference lists and citations of the 12 retrieved full‐text. The reason for excluding five articles were described in Figure 1. The quality of the four studies was assessed by RoB, four studies by JBI tool, and two studies by NIH tool. No study was excluded based on the quality assessments, which finally left 10 studies with a total sample No. 1348, for the systematic review (Table 1).

**Table 1:** Characteristic of reviewed studies (*n* = 10).

Ref. No.	Study author (year)	Study design	Setting	Sample size	PIP program and components, Intervention (I)/Control group (C.)	Result and conclusion	ROB/JBI/NIH
[14]	Heo et al.^14^	Randomized double blind, prospective study	Ilsan Paik Hospital	Total: 45 IG: 22 CG: 23	• I: Donut‐shaped cushion • C: Hydrophilic foam (control group) (20 cm × 20 cm × 0.5 cm	Patients who underwent open‐heart surgery for more than three hours and used a donut‐shaped cushion did not develop pressure injuries, although no statistical difference was noted (*p* = 0.083).	ROB: low risk
[15]	Madeira^15^	A single group, pre‐ and post‐ intervention EBP design	Cardiova‐scular operating room (CVOR) and intensive care unit (CVSICU) at the Johns Hopkins Hospital	23	• I: 1. Preoperative screen for all patients before surgery or on admission to the CVSICU. 2. Implement a process where patients recover on a rental air‐fluidized specialty bed. 3. Standardize PI prevention utilizing a bedside reference tool. 4. Educate OR and CVSICU staff on PIP strategies, including the correct placement of sacral and pressure‐point preventative dressings.	overall improvement in the UAPI count and incident rate that was found to be statistically significant over the screening process. Chi Square test of 2‐proportions showed a reduced PI incidence of 8.56% (Z = 1.66, *p* = 0.048) and 2‐sample Poisson rate showed significance in count (Z = 1.95, *p* = 0.036). an overall cost savings of $78,660.	NIH: good
[16]	Eberhar‐dt et al.^16^	Intrapatient, parallel, pen, randomised clinical trial	university hospital in southern Brazil	135 patients/270 heels IG: 135 heels CG: 135 heels cardiac surgery: *n* = 91	• I: Multilayered silicone foam consists of a soft silicone wound contact layer (Safetac) on a polyurethane film carrier; a flexible, absorbent pad in three layers: a polyurethane foam, a viscose/polyester non‐woven spreading layer and a layer with super absorbent polyacrylate fibers; an outer polyurethane film, which is vapor permeable and waterproof C: transparent polyurethane film	The pressure injury incidence was significantly lower in the intervention group (26.7%), compared with the control group (*p* = 0.001); relative risk of 0.57. Multi‐layered silicone foam (intervention) is more efficacious than transparent polyurethane film (control) in the prevention of pressure injuries caused by surgical positioning of individuals undergoing elective cardiac and gastrointestinal surgery.	ROB: some concern
[17]	Li et al.^17^	Clinical trial	Xinjiang Medical university	Total: 161 IG: 82 CG: 79	• I: Routine nursing and care bundle include skin protection (Special skin care included evaluation of pressure ulcers, laying cotton pads, applying colloids, adhering dressings, moderate lifting, dynamic inspection and handover inspection and restoring normal body temperature. Foam dressings were applied to the head, shoulders, sacral tail and heels that are prone to pressure sores. If possible, patients were gently lifted for 10 min at an interval of about 1 h. • C: routine nursing (A foam dressing was applied for skin protection according to the condition of the patient and the operation time.)	The intraoperative pressure ulcers in the care bundle group (4.9%) were significantly lower than control group (16.5%) (*p* < 0.05). Then care bundle is beneficial to nursing care of Aortic Dissection (AD) surgical treatment and can effectively promote recovery of AD patients.	JBI: 7/9

Ref. No.	Study author (year)	Study design	Setting	Sample size	PIP program and components, Intervention (I)/Control group (C.)	Conclusion	ROB/JBI/NIH
[18]	Strauss et al.^18^	uncontrolled before‐and‐after quality improvement method 21	Single urban academic medical center	Preinterve‐ntion, *n* = 300 postinterve‐ntion, *n* = 224	• I: Placement of a prophylactic 9 × 9 ‐cm anisotropic multilayer silicone foam dressing (Mepilex Border Sacrum; Mö lnlycke Healthcare AB, Gothenburg, Sweden) on the patient's sacrumprior to surgery.	The use of silicone foam dressings may be an effective prophylactic intervention to reduce the incidence of perioperative deep‐tissue pressure injuries among cardiac surgery patients (*p* < 0.02). An overall cost saving of $1,435,728 annually.	NIH: Fair
[19]	Huang et al.^19^	Quasiexperimental, prospective randomized control trial	Yuhuangding Hospital, Yantai, China. 22‐bed surgery department.	Total: 120 IG: 60 CG: 60	• I: Alternative inflatable head pad (with a maximum height of 5–6 cm) was positioned under the patient's head and each section was inflated sequentially every 30 min over the course of the operation. • C: gel head pad (patients being repositioned every hour during surgery in both groups)	The alternating inflatable head pad was effective in reducing the incidence and severity of occipital pressure ulcer and alopecia associated with surgery. (9 cases/60 cases vs. 1 case/60 cases, *p* < 0.01).	ROB: some concern
[20]	Brindle and Wegelin,^20^	Nonrandomized, quasi‐experimental prospective cohort	CSICU at Virginia Commonwe alth university	Total: 85 IG: 50 CG: 35	• I: Standard preventive care plus application of the silicone border foam dressing. (18×8 cm and 23×23 cm) according to body habitus • C: Standard preventive care included placement on a low air loss bed	No statistically significant difference in pressure ulcer incidence between the intervention and control groups was found (*p* = 0.058).	JBI: 8/9
[21]	Feuchti‐nger et al.^21^	Randomized Controlled Trial	Cardiova‐scular Surgery Dep. university Hospital, Freiburg	Total: 175 IG: 85 CG: 90	• I: The test OR table configuration (OR table with waterfilled warming mattress and a 4‐cm thermoactive viscoelastic foam overlay) • C: the standard OR table configuration (OR table with waterfilled warming mattress)	The combination of a 4‐cm viscoelastic foam overlay and a warming source cannot be recommended for pressure ulcer prevention on the operating room table.	ROB: some concern
[22]	Gray et al.^22^	Clinical Trial	–	44	• I: The foam mattresses, Softform Premier. These mattresses have a two‐way stretch polyurethane cover, a contoured foam insert pad and a U‐shaped foam pad. A toughened polyurethane base covers the base of the foam pad. These mattresses were placed on Softform Proflex. The bed frame is in a four‐section profiling frame with a sliding back section. Features of the frame include a height variation of 48 ‐ 85.5 cm, a 17.7 kg stone.	The performance of the Softform Premier and the proflex electric bed frame in this clinical trial indicate that the high/very high‐risk subjects recovering from major surgery were managed effectively without tissue damage.	JBI: 5/9 (due to not having control group)
[23]	Jesurum et al.^23^	Quasi experimental design	Teaching hospital in south central United State.	Total: 36 IG: 16 CG: 20	• I: Low‐air loss bed that have 16 compartmentalized air sacs • C: Standard beds with a pressure‐reducing polyurethane foam that is covered by a waterproof fabric to reduce the potential of liquid accumulation inside the mattress.	The result suggests that LAL therapy does not make a difference in the prevention of pressure ulcers.	JBI: 7/9

Abbreviations: C, control; CG, control group; I, intervention; IG, intervention group.

### 3.2 Included study characteristics

The 10 included studies’ characterization are described in Table 1. Regarding the PI preventive interventions, most of the featured studies focused on multilayer silicone foam.^16^, ^18^, ^20^ Other studies were about implementation of donut‑shaped cushions,^14^ Care bundles including skin protection^17^ multiple intervention programs including educating operating room and cardiovascular surgical intensive care unit staff on PIP strategies,^15^ alternative inflatable head pads,^19^ thermoactive viscoelastic foam pad,^21^ foam mattresses and electric bed frames,^22^ and low‐air loss bed.^23^ There was inadequate data about the effectiveness of other PI preventive strategies and interventions such as mobilization and repositioning, creams, and nutritional supplements.

### 3.3 Main findings

Patient outcomes reported included PI incidence and prevalence and the binary outcome was assignment to intervention versus standard care and some other equipment or programs. In 6 of the 10 research studies, the PI prevention interventions resulted in significant decreases in PI cumulative incidence^15^, ^16^, ^17^, ^18^, ^19^ ^,^ ^22^; and a decrease in PI cumulative incidence with no statistical significance reported in four studies^14^ ^,^ ^20^ ^,^ ^21^ ^,^ ^23^; two studies reported estimated reduced costs, with one reported savings of $78,660 after the implementation focused on Multiple intervention programs,^15^ and another with a saving of $1,435,728 annually after the placement of a prophylactic anisotropic multilayer silicone foam dressing on the patient's sacrum before surgery.^18^ The present systematic review identified four domains in terms of PI prevention among the included studies: multilayer silicone foam, Care bundle and multiple intervention programs, alternative inflatable head pads, pressure‐reducing foam mattresses, and electric bed frames.

#### 3.3.1 Multilayer silicone foam

There are evidence that the use multilayered silicone foam can reduce the incidence of PI, in addition to reducing costs, because it has the ability to reduce pressure forces and friction, as well as transferring shear away from critical areas.^6^, ^7^, ^8^ Among three included studies that assessed multilayer silicone foam as a PI preventive measure, two studies showed a statistically significant reduction in PI incidence rates.

#### 3.3.2 Care bundle multiple intervention programs

care bundles and multiple intervention programs are a set of evidence‐based interventions that when performed together had a better and positive impact on patient outcomes when compared with individual interventions.^24^ Two included studies with implementation multiple intervention program were effective in decreasing the incidence of PIs.^15^, ^17^

#### 3.3.3 Alternative inflatable head pad

An Alternating Pressure Air Mattresses (APAMs) could decrease the incidence of pressure lesions via repositioning to reduce the duration of direct pressure and shearing force.^25^

#### 3.3.4 Pressure reducing foam mattress and electric bed frames

Mattresses, overlays, and cushions made of high‐density or contoured foam or filled with beads, gel, fiber, air, or water that increase the area of contact between the patient and the support surface and thus reduce the pressure at the interface.^26^ Pressure‐reducing mattresses are often seen as central to any coherent PI prevention plan.^27^ Another development was the advent of electric bed frames, where different parts of the frame can be adjusted remotely to help the patient movement. These electric bed frames can be used in conjunction with pressure‐reducing foam mattress and also the visco‐elastic foam mattress. These mattresses mold to the patients’ body, thereby maximizing pressure redistribution.^28^

## 4 DISCUSSION

Using the PRISMA statement for study design, this review found 10 studies that evaluated different PI prevention interventions and strategies. In the study of Heo et al., PI of NPUAP stage I or higher developed in three patients underwent open heart surgery (13%), and all were in the control group and patients who used a donut‑shaped cushion did not develop pressure injuries, but there was no significant difference (*p* = 0.083).^14^ using a hydrophilic foam dressing as a control might have decreased the PI incidence.

Eberhardt et al. reported that multilayered silicone foam is more efficacious than transparent polyurethane film in the prevention of PIs caused by surgical positioning.^16^ Also, Strauss et al. study result showed that multilayer silicone foam is an effective prophylactic intervention to reduce the incidence of perioperative deep‐tissue PIs among cardiac surgery patients and it also is a cost‐effective strategy used to prevent PI.^18^ Cornish L. suggested that the use multi‐layered silicone foam can reduce the incidence of PI, in addition to reducing costs.^6^ A study that aimed to evaluate the clinical efficacy of using multi‐layered silicone foam compared with polyurethane film in the prevention of PI in the intraoperative period of the spine showed some similar results.^29^ Also in the OR, using a five‑layer silicone sacral foam dressing for elective vascular surgical cases resulted in a significant decrease in OR‑related sacral PIs in study of Riemenschneider KJ.^30^ Similar to these finding, a systematic review conducted by Tayyib and Coyer revealed the effectiveness of using silicon foam dressing for preventing sacral HAPUs in ICU settings.^31^ Hence, multilayered silicone foam may be considered as the standard in PI prevention. However Brindle et al. found that there is no statistically significant difference in PI incidence between the patients who received standard preventive care plus application of the silicone border foam dressing compared with those who received standard preventive care included placement on a low air loss bed.^20^

The specialist nursing team in Li et al. study implemented a special model for the prevention of pressure ulcers, containing seven elements for skin protection, as a part of care bundle, include laying cotton pads, applying colloids, adhering dressings, moderate lifting, dynamic inspection and handover inspection and restoring normal body temperature, applying foam dressings to the head, shoulders, sacral tail and heels that are prone to pressure sores and lifting patients gently for 10 min at an interval of about 1 h; and consistent with previous studies,^32^, ^33^ Li mentioned that the intraoperative pressure ulcer rate in the care bundle group was significantly lower than control group, indicating that this skin protection model was effective in preventing Type A Aortic Dissection operative pressure ulcers and could significantly reduce the incidence of pressure ulcers, thereby reducing postoperative complications.^17^

Although due to focus on intraoperative patients positioning, Beckett A.E. mentioned that complicated surgery performed over several hours with inadequate padding and incorrect positioning can cause serious and long‐term injury^34^ and Nilsson UG. considered that intraoperative positioning of patients plays a crucial role in the prevention of PIs,^35^ in our study, evidence was inadequate to reliably determine the effectiveness of intraoperative patients repositioning.

Gray et al. evaluated the combination of a pressure‐reducing mattress with an electric profiling bed frame and two outcome measures were used in his study: pressure ulcer incidence and perceptions of comfort that No pressure ulcers developed in patients and the perceptions of comfort were generally positive.^22^ Hampton study result also identified that patients experienced greater comfort on beds with the electric facility, produced less pressure sores, mobilized easily and pressure sore prevention costs could be reduced.^36^

## 5 LIMITATION

First, regarding difference between intervention and strategies used to PIP, we could not conduct a meta‐analysis. Secondly, in defining intervention groups, we disregarded confounded co‐interventions of included studies (e.g., repositioning) because these co‐interventions were received equally by patients in both intervention and control group and they might have been influenced the effectiveness of main interventions.

## 6 CONCLUSION

The present review demonstrated different prevention strategies with significant decrease in PI cumulative incidence and prevalence in patients undergoing open heart surgery. According to current evidence, multilayer silicone foam, Care bundle and multiple intervention programs, alternative inflatable head pad, pressure reducing foam mattress, and electric bed frames are effective strategies to prevent pressure ulcers and according to our recent review the gender (females more than males), advanced age, diabetes, surgery duration, and preoperative serum albumin level are as the risk factors for developing PI in cardiac surgical patient that it needs to consider these risk factors to prevent PI before and during surgery. Further studies are needed to specify the cost‐effectiveness of mentioned strategies and RCTs to investigate other PIP strategies such as repositioning and mobilization, nutritional supplements, creams, and the co‐interventions effect.

## AUTHOR CONTRIBUTIONS

**Hamed Taghiloo**: Investigation; methodology; supervision; validation; writing—review and editing. **Abbas Ebadi**: Conceptualization; methodology; supervision; validation; writing—review and editing. **Yaser Saeid**: Conceptualization; investigation; methodology; supervision; validation; writing—review and editing. **Alireza Jalali Farahni**: Methodology; validation; writing —review and editing. **Atefeh Davoudian**: Formal analysis; investigation; methodology; resources; writing—original draft.

## CONFLICT OF INTEREST STATEMENT

The authors declare no conflict of interest.

## TRANSPARENCY STATEMENT

The lead author Yaser Saeid affirms that this manuscript is an honest, accurate, and transparent account of the study being reported; that no important aspects of the study have been omitted; and that any discrepancies from the study as planned (and, if relevant, registered) have been explained.

## Data Availability

The data that support the findings of this study are available from the corresponding author upon reasonable request.
